# Clinical and epidemiological profile of hospitalized patients with leptospirosis: a cross-sectional study in Durán, Ecuador, 2023

**DOI:** 10.3389/fcimb.2026.1921311

**Published:** 2026-09-23

**Authors:** Diego Edilberto Pinza Vera, Sandra Chica Jácome, Juan S. Izquierdo-Condoy, Jorge Vasconez-Gonzalez, Esteban Ortiz-Prado

**Affiliations:** 1 Departamento de Medicina Interna, Hospital General Dr. Enrique Ortega Moreira, Durán, Ecuador; 2 One Health Research Group, Faculty of Health Sciences, Universidad de Las Américas, Quito, Ecuador

**Keywords:** clinical epidemiology, co-infection, dengue, Ecuador, leptospirosis

## Abstract

**Introduction:**

Leptospirosis is a globally distributed zoonosis, particularly prevalent in tropical and subtropical regions, with epidemic potential during the rainy season. It is caused by pathogenic Leptospira species and produces a wide clinical spectrum, ranging from mild febrile illness to severe, life-threatening disease.

**Objective:**

To describe the clinical and epidemiological characteristics of hospitalized patients with serologically supported leptospirosis in Durán, Guayas province, Ecuador.

**Methods:**

A descriptive, cross-sectional study was conducted at the Hospital General Dr. Enrique Ortega Moreira between January and June 2023. Patients aged 15 years or older with fever and positive IgM serology for leptospirosis were included. Data were obtained from anonymized medical records. Categorical variables were summarized as frequencies and percentages, and continuous variables as medians with interquartile ranges (IQR). Group comparisons used the Pearson chi-square or Fisher exact test for categorical variables and the Mann–Whitney U test for continuous variables.

**Results:**

Forty-eight patients met the inclusion criteria. Most were male (58.3%) and aged 15–39 years (85.5%). The most frequent symptoms were fever (93.8%) and diarrhea (83.3%). The median duration of fever was 17 days (IQR 8–21), reaching up to 60 days. Concurrent dengue IgM positivity was identified in 22.9% of patients and was associated with a longer median fever duration (19 vs 16 days; p=0.036). Frequent laboratory abnormalities included hypoalbuminemia (86.4%), hyponatremia (82.9%), hypokalemia (55.3%), thrombocytopenia (54.2%), and anemia (54.2%). The median hospital stay was 7 days (IQR 5–8).

**Conclusions:**

Hospitalized leptospirosis cases in Durán during the 2023 rainy season showed frequent gastrointestinal manifestations, laboratory abnormalities, prolonged fever, and substantial dengue IgM positivity. These findings highlight diagnostic challenges in endemic coastal settings and support the need for strengthened surveillance, systematic evaluation of co-circulating infections, and national clinical practice guidelines for leptospirosis.

## 1 Introduction

Leptospirosis is a zoonotic disease of global distribution, particularly prevalent in tropical and subtropical regions, with seasonal peaks during the rainy season; it is recognized as a neglected communicable disease with epidemic potential (World Health Organization, 2011; Haake and Levett, 2015). It is caused by spirochetes of the genus Leptospira, for which 21 species have been described according to genotypic classification, nine of them pathogenic (*L. interrogans, L. kirschneri, L. noguchii, L. borgpetersenii, L. weilii, L. santarosai, L. alexanderi, L. kmetyi, and L. alstonii*) (Rajapakse, 2022; Calvopiña et al., 2023). Humans typically acquire the infection through direct contact with the urine of infected animals or with environments contaminated with such urine (**Figure 1**). Because of this transmission pathway, leptospirosis predominantly affects occupational groups with frequent animal or environmental exposure, such as ranchers, farmers, veterinarians, and sewage workers (Haake and Levett, 2015; Calvopiña et al., 2023).

**Figure 1:**
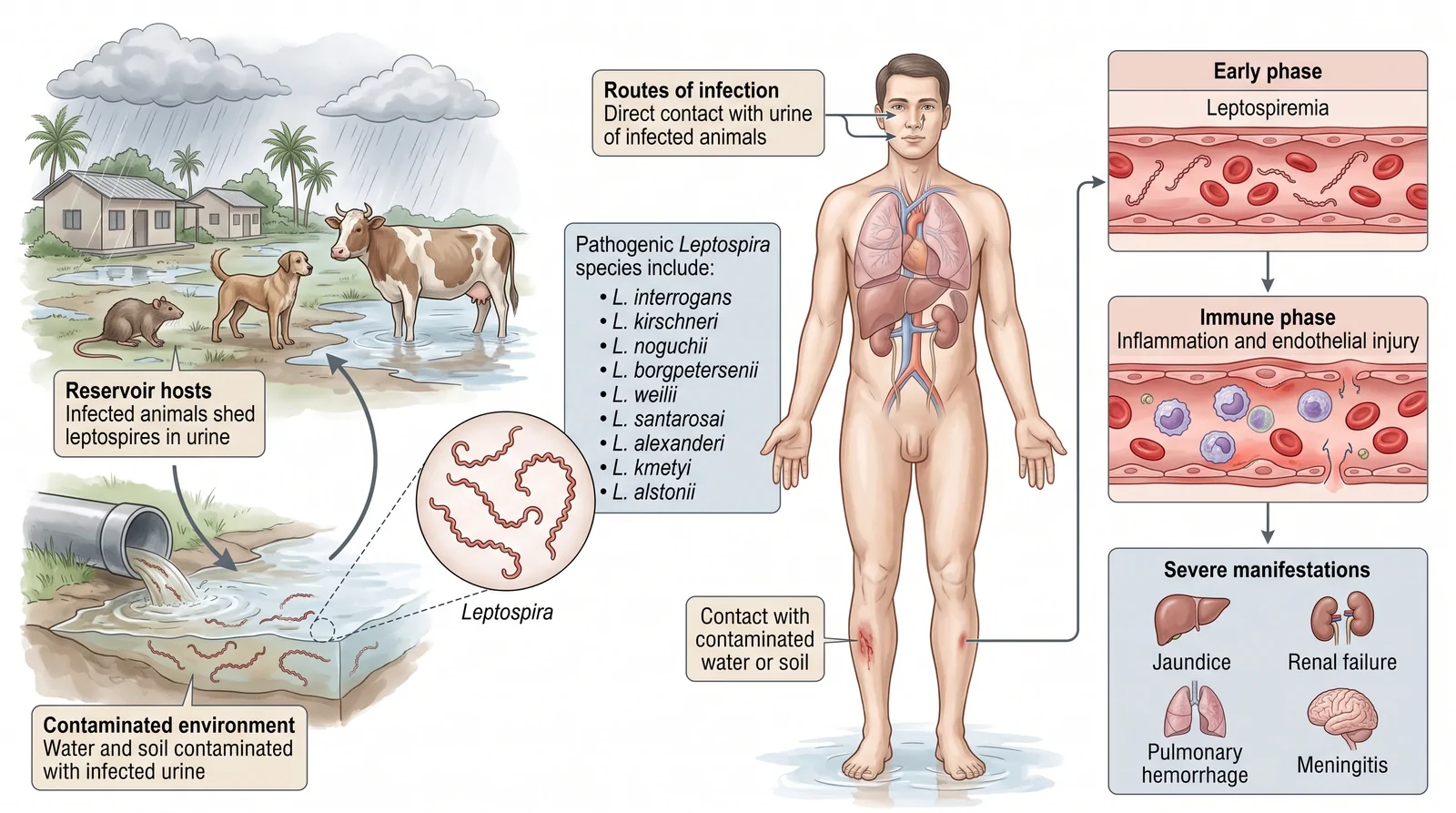
Transmission cycle of Leptospira spp., showing reservoir hosts, environmental contamination, routes of human infection, and the principal clinical phases of disease.

Clinically, leptospirosis is highly variable, ranging from mild, self-limiting illness to severe and occasionally fatal disease. The incubation period is usually 5–14 days (range 2–30 days) (Johnson, 1996). In the acute phase, patients frequently present with high fever, myalgia, and frontal or retro-orbital headache, often accompanied by nausea, vomiting, abdominal pain, diarrhea, cough, photophobia, and a rash on the trunk or pretibial region (Johnson, 1996). In the immune phase, manifestations may include prolonged fever, jaundice, acute kidney injury, pulmonary hemorrhage, respiratory failure, hypotension, myocarditis, meningitis, and altered mental status (Johnson, 1996).

Globally, an estimated 73% of cases occur in tropical regions such as Southeast Asia, eastern sub-Saharan Africa, the Caribbean, and Oceania (Costa et al., 2015). Leptospirosis causes approximately 1 million cases and more than 50, 000 deaths annually (Costa et al., 2015) with a global burden of 2.90 million disability-adjusted life years (DALYs) (Torgerson et al., 2015). In South America, the Pan American Health Organization reports an estimated 10, 702 cases per year, with Brazil contributing 38% of cases, followed by Peru (22%), Colombia (8%), Ecuador (7%), and Costa Rica (4%) (Schneider et al., ).

In Ecuador, the estimated annual incidence is approximately 1 case per 100, 000 inhabitants (Calvopiña et al., 2023). Núñez-González et al. reported a decline in incidence from 3.3 to 0.8 cases per 100, 000 inhabitants between 2013 and 2018 (Núñez-González et al., 2020). However, more recent data from the national surveillance system (SIVE-ALERTA) indicate a marked resurgence: 134 cases in 2022, 663 in 2023, 595 in 2024, and a record 1, 373 in 2025 (Ministerio de Salud Pública Ecuador, 2024). Despite this epidemiological increase, published studies addressing the clinical and epidemiological profile of leptospirosis in Ecuador remain strikingly scarce. This knowledge gap hinders an adequate understanding of disease presentation in the local context, where ecological and socioeconomic conditions may shape distinct epidemiological patterns.

Therefore, the aim of this study was to describe the clinical and epidemiological characteristics of hospitalized patients with serologically supported leptospirosis diagnosed at a public hospital in Durán, Ecuador.

## 2 Methodology

### 2.1 Study design

A retrospective, descriptive, cross-sectional observational study was conducted using medical records of patients hospitalized at the Hospital General Dr. Enrique Ortega Moreira, Durán, Ecuador, between 1 January and 30 June 2023. The study period corresponds to the dates of the clinical encounters included in the cohort rather than to prospective research enrollment. The study was subsequently developed through retrospective review of existing medical records after recognition of an unusual pattern of persistent febrile illness during the 2023 outbreak period.

### 2.2 Setting and population

Ecuador, on the northwestern coast of South America, is administratively divided into 24 provinces and four geographical regions: the Coast, the Andes, the Amazon, and the Galápagos Islands. The national health system comprises three sectors—social security, the private sector, and the public sector—the latter providing universal free healthcare (Instituto Nacional de Estadística y Censos, 2022).

Durán is a coastal canton in Guayas province, with a population of 303, 910 inhabitants according to the 2022 national census (Instituto Nacional de Estadística y Censos, 2024). It lies on the eastern bank of the Guayas River, adjacent to Guayaquil, and is characterized by a warm, humid climate, fertile lowlands, and substantial commercial and industrial activity. These environmental and socioeconomic conditions, together with high vehicular density and rapid urban expansion, create a favorable setting for the transmission of zoonotic and waterborne diseases such as leptospirosis. The study population consisted of patients hospitalized with suspected leptospirosis at this hospital during the study period. As the primary public facility serving Durán and surrounding areas, it plays a pivotal role in the epidemiological surveillance of infectious diseases in the region.

### 2.3 Selection criteria

Patients were eligible if they were 15 years of age or older, had clinically suspected leptospirosis with current or recent febrile illness, and had positive anti-*Leptospira* IgM serology. Only patients hospitalized during the study period (January–June 2023) were considered. Patients under 15 years of age, cases with compatible symptoms but negative serology, and diagnoses established outside the predefined timeframe were excluded.

### 2.4 Data collection

Clinical data were obtained through review of the hospital’s anonymized medical records. Non-probabilistic consecutive sampling was applied, and all eligible records meeting the inclusion criteria were analyzed. The final sample comprised patients with clinically suspected leptospirosis and positive anti-*Leptospira* IgM serology admitted during the specified period.

Anti-*Leptospira* IgM antibodies were assessed in serum at the National Institute for Public Health Research (INSPI), Ecuador’s national reference laboratory. Samples collected at the hospital were referred to INSPI for processing. According to the national diagnostic procedures used by INSPI, anti-*Leptospira* IgM antibodies are detected using an indirect enzyme-linked immunosorbent assay (ELISA). The specific commercial assay used for the samples included in this retrospective cohort was not available in the medical records.

Dengue IgM testing was performed at the Hospital General Dr. Enrique Ortega Moreira. The test performed was the Dengue IgG/IgM Rapid Test Cassette, a rapid immunochromatographic assay designed to detect IgG and IgM antibodies against the four dengue virus serotypes in blood, serum, or plasma samples It is important to note that, at the time of patient admission, the hospital did not have access to NS1 antigen testing. Dengue and anti-*Leptospira* IgM testing were not necessarily performed on the same date. In some patients, dengue was initially considered during evaluation of the febrile illness, whereas persistence of fever subsequently prompted testing for leptospirosis. Therefore, the two serological results did not necessarily correspond to samples obtained at the same time point. No repeat serology, paired-serum testing, microscopic agglutination testing (MAT), PCR, or other confirmatory testing was performed in patients with concurrent dengue and anti-*Leptospira* IgM positivity.

Malaria testing was not routinely performed because no epidemiological exposure or clinical indication prompting investigation for malaria was documented by the treating teams.

A standardized mild, moderate, or severe leptospirosis classification had not been prospectively assigned in the original clinical records. To avoid potential *post hoc* misclassification, patients were therefore not retrospectively categorized according to disease severity; instead, available clinical manifestations, laboratory abnormalities, organ dysfunction, supportive interventions, and outcomes were described individually.

### 2.5 Statistical analysis

Categorical variables were summarized as frequencies and percentages, and continuous variables as medians with interquartile ranges (IQR). For variables not measured in all patients, percentages were calculated using the number of tested patients as the denominator, as indicated in the corresponding table footnotes. Associations between categorical variables (e.g., sex and clinical manifestations) were assessed with the Pearson chi-square test or the Fisher exact test, as appropriate. Differences in continuous variables between groups—for example, fever duration and length of hospital stay according to dengue IgM status—were evaluated with the Mann–Whitney U test. A two-sided p value < 0.05 was considered statistically significant. Analyses were performed in IBM SPSS Statistics version 24.0 (IBM Corp., Armonk, NY, USA).

### 2.6 Ethical considerations

The study was approved by the Ethics Committee for Research in Human Beings of Universidad de Las Américas (CEISH-UDLA), approval code 2025-OBS-019. Authorization to access the clinical records was granted by the Directorate of Teaching of the Hospital General Dr. Enrique Ortega Moreira, Durán, Ecuador. Only two patients included in the study were minors, both aged 15 years. Clinical information was obtained exclusively from anonymized medical records, and no direct research-related interaction with patients occurred. All procedures were conducted in accordance with the principles of the Declaration of Helsinki.

## 3 Results

### 3.1 Demographic characteristics

A total of 48 patients with febrile illness and positive IgM serology for leptospirosis were hospitalized between 1 January and 30 June 2023. Most were men (58.3%, n=28), and women accounted for 41.7% (n=20) (**Table 1**). Most patients were aged 15–39 years (85.5%). The most frequently recorded occupations were homemaker (29.2%) and student (25.0%), followed by manual laborer (10.4%). At least one comorbidity was documented in 15 patients (31.3%); the most frequent were bronchial asthma and hepatic cirrhosis (each n=2), with isolated cases of diabetes mellitus, chronic kidney disease, and other conditions.

**Table 1:** Demographic characteristics of hospitalized patients with leptospirosis (n=48).

Characteristic	Category	n	%
Sex	Male	28	58.3
	Female	20	41.7
Age(years)	15–39	41	85.5
	40–63	3	6.3
	>63	4	8.3
Occupation	Homemaker	14	29.2
	Student	12	25.0
	None	9	18.8
	Manual laborer	5	10.4
	Agronomist	1	2.1
	Cardboard-factory worker	1	2.1
	Driver	1	2.1
	Businessperson	1	2.1
	Mechanic	1	2.1
	Machinery operator	1	2.1
	Teacher	1	2.1
	Supervisor	1	2.1

Percentages are calculated over the total sample (n=48).

### 3.2 Clinical manifestations

The most frequent symptom was fever (93.8%), followed by diarrhea (83.3%), abdominal pain (77.1%), nausea or vomiting (72.9%), headache (66.7%), and chills (60.4%). Less frequent manifestations included gastrointestinal bleeding such as melena or hematochezia (18.8%), odynophagia (14.6%), cough (10.4%), and gingivorrhagia or epistaxis (8.3%) (**Table 2**). The only symptoms that differed significantly by sex were diarrhea, which was more frequent in men (26/28 vs 14/20; p = 0.036), and arthralgia, which was more frequent in women (13/20 vs 9/28; p = 0.024). One patient (2.1%) developed neurological complications presenting with headache and progressive deterioration of consciousness, additionally, the patient required mechanical ventilation. and subsequently died during hospitalization, corresponding to an overall in-hospital mortality of 2.1% (1/48).

**Table 2:** Signs and symptoms in hospitalized patients with leptospirosis and their distribution by sex (n=48).

Sign/ symptom	n	%	Male	Female	p value
Fever	45	93.8	25	20	0.131
Diarrhea	40	83.3	26	14	**0.036**
Abdominal pain	37	77.1	20	17	0.270
Nausea/vomiting	35	72.9	20	15	0.784
Headache	32	66.7	16	16	0.098
Chills	29	60.4	16	13	0.583
Dehydration	22	45.8	13	9	0.922
Arthralgia	22	45.8	9	13	**0.024**
Myalgia	19	39.6	10	9	0.517
Anorexia	20	41.7	13	7	0.428
Jaundice	13	27.1	8	5	0.784
Hematochezia/melena	9	18.8	5	4	0.851
Odynophagia	7	14.6	3	4	0.369
Cough	5	10.4	3	2	0.936
Gingivorrhagia/epistaxis	4	8.3	2	2	0.724

Values are counts of patients presenting each sign or symptom. Percentages are over the total sample (n=48). p values were obtained with the Pearson chi-square test comparing presence of the symptom by sex; values < 0.05 are shown in bold. Bold values indicate the only symptoms that differed significantly by sex.

### 3.3 Duration of fever

The median duration of fever was 17 days (IQR 8–21; range 0–60), with a mean of 17.1 days among the 47 patients with available data. Prolonged fever (>15 days) was documented in 26 patients (55.3%), and two male patients had fever lasting more than 45 days, including one case persisting for 60 days. The distribution of fever duration was comparable between men (median 17 days) and women (median 16 days). Patients were generally admitted around the fifth day of fever, consistent with the typical clinical course of dengue with warning signs. Fever duration prior to hospitalization was obtained from the patients’ medical history and documented in the medical records, while fever occurring during hospitalization was monitored through daily clinical assessment (**Figure 2**).

**Figure 2:**
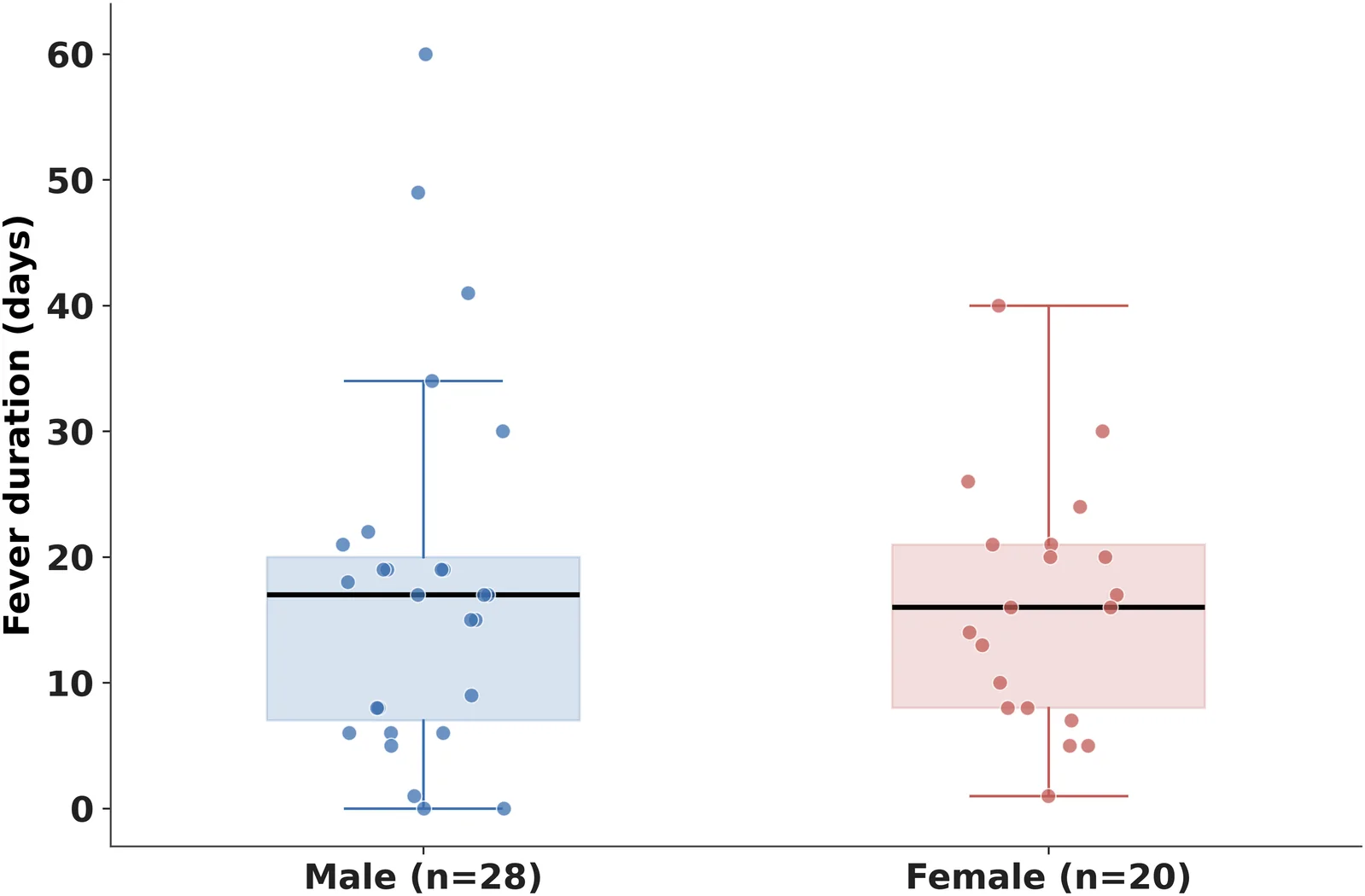
Distribution of fever duration (days) by sex. Boxes show the median and interquartile range; whiskers extend to the most extreme values within 1.5 × IQR, and individual patients are shown as points. Fever duration was available for 47 of 48 patients (one male value missing).

### 3.4 Temporal distribution of admissions

No cases were admitted in January 2023. Admissions rose progressively from February (n=6) to a peak in April (n=18) and then declined through May and June (n=6 each). March and April together accounted for 30 of the 48 cases (62.5%), indicating a clear temporal clustering that coincided with the peak of the coastal rainy season and the El Niño–related flooding of early 2023.

### 3.5 Laboratory findings

Concurrent dengue IgM positivity was identified in 11 patients (22.9% of the full cohort and 26.8% of the 41 patients tested). Laboratory abnormalities were frequent and included hyponatremia (82.9% of tested), hypoalbuminemia (86.4% of tested), thrombocytopenia (54.2%), anemia (54.2%), and hypokalemia (55.3%). Severe thrombocytopenia (<50, 000/µL) was present in 7 patients, corresponding to 26.9% of those with thrombocytopenia and 14.6% of the cohort. Prolongation of the international normalized ratio (INR) occurred in 67.5% of tested patients, elevated creatinine in 31.9%, and any degree of hematuria in 56.3% (microscopic blood +, 37.5%; ++, 8.3%; +++, 10.4%). (**Figure 3**).

**Figure 3:**
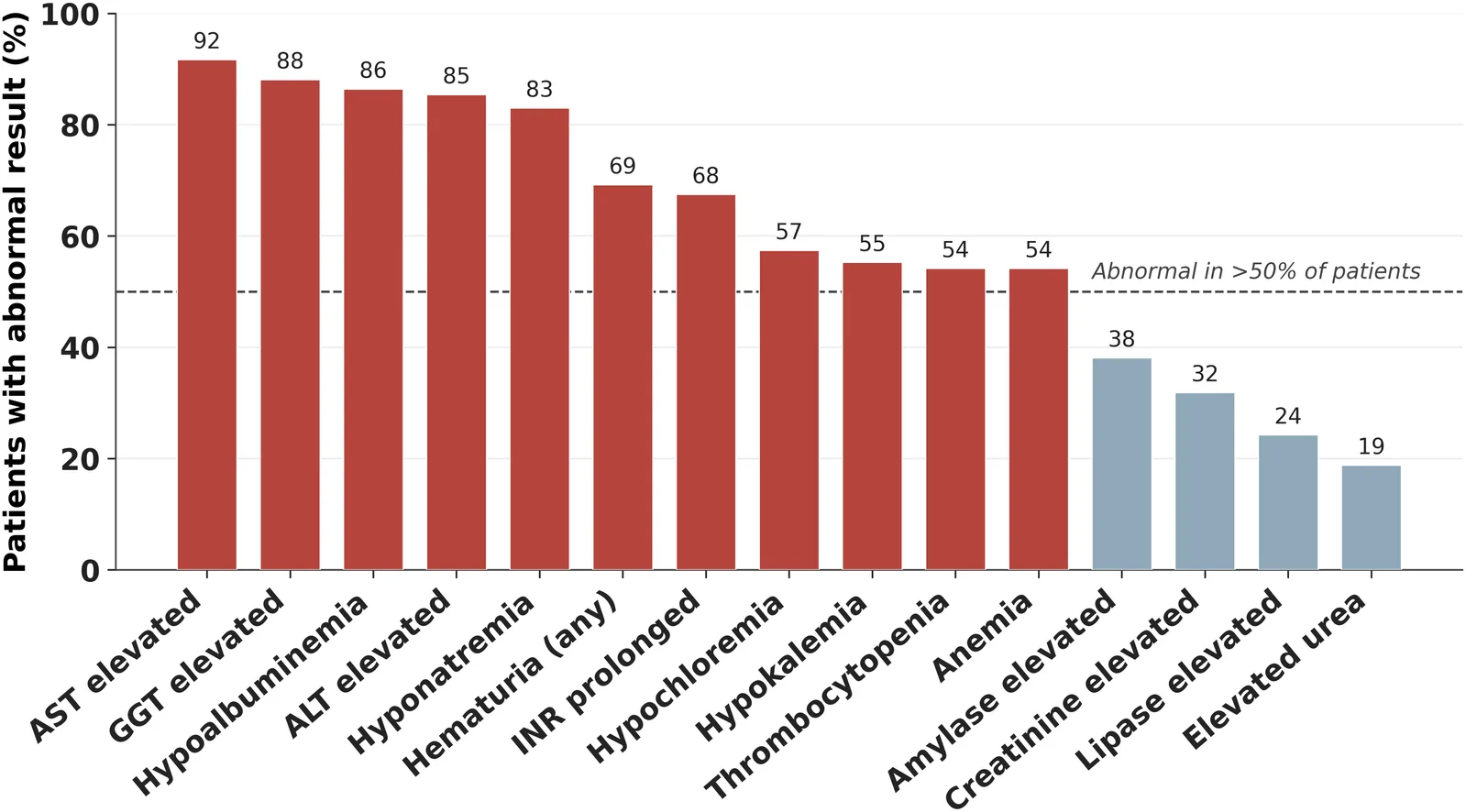
Frequency of laboratory abnormalities among hospitalized patients with leptospirosis. Bars show the percentage of tested patients with an abnormal result for each parameter, ranked in descending order; the dashed line marks the 50% threshold, and abnormalities present in more than half of patients are highlighted in red. Denominators correspond to the number of patients tested for each analyte. ALT, alanine aminotransferase; AST, aspartate aminotransferase; GGT, gamma-glutamyl transferase; INR, international normalized ratio.

Liver-function tests were frequently altered, with elevations in aminotransferases (ALT and AST), gamma-glutamyl transferase, alkaline phosphatase, and total bilirubin. Amylase and lipase were elevated in 33.3% and 27.1% of patients, respectively. The overall spectrum and frequency of abnormalities are summarized in **Figure 3** and detailed in **Table 3**.

**Table 3:** Laboratory findings in hospitalized patients with leptospirosis (n=48).

Test	Category	n	%
Dengue serology	IgM positive	11	22.9
	IgM negative	30	62.5
	Not tested	7	14.6
Hemoglobin	<7 g/dL	2	4.2
	7.0–10.0 g/dL	12	25.0
	10.1–10.9 g/dL	12	25.0
	11.0–16.0 g/dL	22	45.8
INR	<1.20	13	27.1
	1.20–2.20	27	56.3
	Not tested	8	16.7
Platelets	<50, 000/µL	7	14.6
	50, 000–99, 000/µL	9	18.8
	100, 000–150, 000/µL	10	20.8
	150, 000–400, 000/µL	22	45.8
Potassium	2.5–<3.0 mEq/L	7	14.6
	3.0–3.5 mEq/L	19	39.6
	3.7–5.2 mEq/L	21	43.8
Sodium	<125 mmol/L	1	2.1
	125–129 mmol/L	11	22.9
	130–135 mmol/L	27	56.3
	135–145 mmol/L	8	16.7
Chloride	<98.0 mEq/L	27	56.3
	98.0–107.9 mEq/L	20	41.7
Urea	<55.0 mg/dL	39	81.3
	>55.0 mg/dL	9	18.8
Creatinine	<1.20 mg/dL	32	66.7
	1.21–3.20 mg/dL	12	25.0
	>3.20 mg/dL	3	6.3
Albumin	≤3.5 g/dL	19	39.6
	>3.5 g/dL	3	6.3
	Not tested	26	54.2
Hematuria	Blood +	18	37.5
	Blood ++	4	8.3
	Blood +++	5	10.4
	No hematuria	12	25.0
	Not tested	9	18.8
Proteinuria	None	9	18.8
	Protein +	16	33.3
	Protein ++	8	16.7
	Protein +++	6	12.5
	Not tested	9	18.8
Ketonuria	Ketones +	9	18.8
	Ketones ++	4	8.3
	Ketones +++	2	4.2
	No ketones	23	47.9
	Not tested	10	20.8
Fecal occult blood	Positive	7	14.6
	Negative	5	10.4
	Not tested	36	75.0
ALT (GPT)	<40 IU/L	7	14.6
	40–<250 IU/L	31	64.6
	250–<400 IU/L	8	16.7
	400–<600 IU/L	1	2.1
	≥600 IU/L	1	2.1
AST (GOT)	<40 IU/L	4	8.3
	40–<250 IU/L	29	60.4
	250–<400 IU/L	6	12.5
	400–<600 IU/L	4	8.3
	≥600 IU/L	5	10.4
GGT	<60 IU/L	5	10.4
	60–<250 IU/L	21	43.8
	250–<400 IU/L	5	10.4
	400–<600 IU/L	5	10.4
	≥600 IU/L	6	12.5
	Not tested	6	12.5
Alkaline phosphatase	120–<250 IU/L	10	20.8
	250–<400 IU/L	13	27.1
	400–<600 IU/L	7	14.6
	>600 IU/L	15	31.3
	Not tested	3	6.3
Total bilirubin	<1.3 mg/dL	27	56.3
	1.3–<2.0 mg/dL	10	20.8
	≥2.0 mg/dL	11	22.9
Amylase	<100 U/L	26	54.2
	100–<250 U/L	12	25.0
	≥250 U/L	4	8.3
	Not tested	6	12.5
Lipase	<60 U/L	24	50.0
	60–<100 U/L	4	8.3
	≥100 U/L	9	18.8
	Not tested	11	22.9

Percentages are calculated over the total sample (n = 48); “Not tested” denotes patients in whom the test was not performed. In the main text and figures, percentages of abnormal results are expressed over the number of patients tested for each analyte.

Among the 22 patients in whom serum albumin was measured, 19 had hypoalbuminemia (≤3.5 g/dL), corresponding to 86.4% of those tested. Proteinuria was also frequent, occurring in 30 of the 39 patients with available urinalysis (76.9%). Hypoalbuminemia therefore occurred in the context of frequent urinary protein loss and liver-related laboratory abnormalities, suggesting that multiple mechanisms may have contributed to reduced serum albumin levels.

Anemia was documented in 26 patients (54.2%), including two patients with hemoglobin levels <7 g/dL. Documented bleeding manifestations included hematochezia or melena in 9 patients (18.8%), gingivorrhagia or epistaxis in 4 (8.3%), and hematuria in 27 of the 39 patients with available urinalysis. Fecal occult blood was positive in 7 of the 12 patients in whom it was assessed. Because a standardized etiological evaluation of anemia was not available, no specific mechanism could be assigned.

Percentages are calculated over the total sample (n = 48); “Not tested” denotes patients in whom the test was not performed. In the main text and figures, percentages of abnormal results are expressed over the number of patients tested for each analyte.

### 3.6 Length of stay and treatment

The median hospital length of stay was 7 days (IQR 5–8; range 0–27). Empirical antibiotic therapy was heterogeneous: ceftriaxone was prescribed in 43 patients (89.6%), alone or in combination, whereas doxycycline was used in 11 patients (22.9%), reflecting the absence of a standardized national treatment protocol. Additionally four patients required red blood cell transfusions.

### 3.7 Concurrent dengue IgM positivity

In an exploratory subgroup analysis, patients with concurrent dengue IgM positivity had a longer median fever duration than dengue IgM-negative patients (19 vs 16 days; Mann–Whitney U, p=0.036) and a non-significantly longer hospital stay (median 9 vs 7 days; p=0.053) (**Figure 4**). **Figure 4** illustrates the distribution of fever duration between both serological groups, showing a shift toward longer febrile periods among patients with dengue IgM positivity.

**Figure 4:**
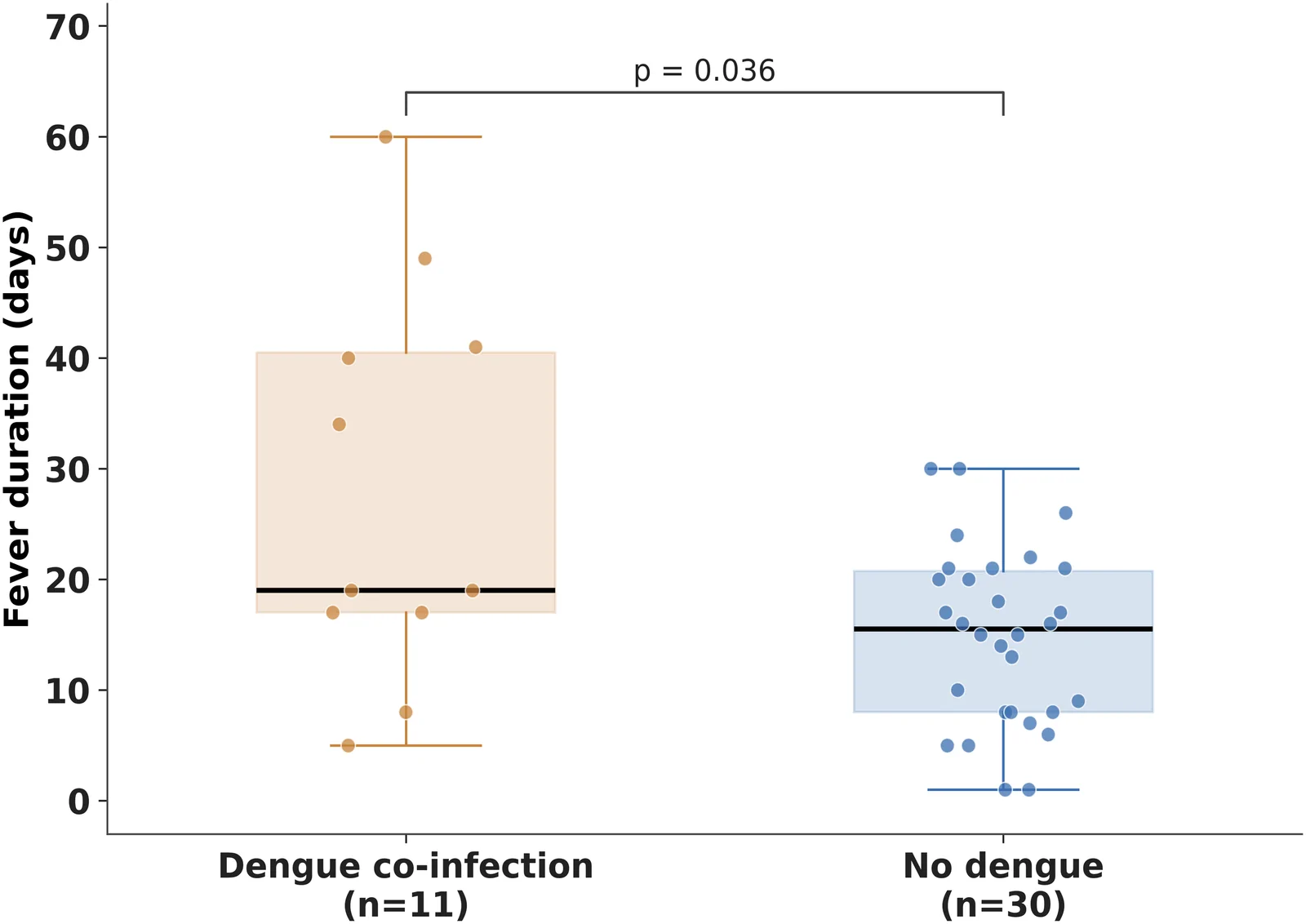
Fever duration according to dengue co-infection. Patients with dengue co-infection (n=11) had a longer median fever duration than those without (n=30) (Mann–Whitney U test, p=0.036). Patients with untested dengue status are not shown.

## 4 Discussion

To our knowledge, this is the first detailed characterization of hospitalized leptospirosis cases in Durán during the 2023 outbreak period. We identified 48 hospitalized patients and characterized their demographic profile, clinical manifestations, laboratory alterations, and concurrent dengue IgM positivity. These findings help fill the knowledge gap regarding the local burden and presentation of leptospirosis in Ecuador, where clinical and epidemiological descriptions remain scarce. Leptospirosis is endemic to Ecuador, with distribution across its four natural regions (Coast, Andes, Galápagos, and Amazon). It affects both humans and animals, and previous studies in the country have confirmed the presence of leptospires in a wide range of species, including opossums, rats, monkeys, rabbits, guinea pigs, dogs, cattle, horses, sheep, pigs, and even sea lions (Baquero et al., 2010; Barragan et al., 2016; Denkinger et al., 2017; Burgos Macias et al., 2019; Orlando et al., 2020).

National surveillance data reveal a sustained resurgence of leptospirosis in Ecuador (**Figure 5**). After an annual average of fewer than 150 notifications between 2015 and 2019, cases rose to 663 in 2023 and, following 595 in 2024, reached a record 1, 373 in 2025—a 130.8% increase over 2024 and more than ten times the 2022 figure (134) (Ministerio de Salud Pública Ecuador, 2024). According to SIVE-ALERTA, this represents an unprecedented surge over historical baseline levels. During 2023, an outbreak with more than 60 reported cases was identified in the provinces of Manabí, Santo Domingo de los Tsáchilas, and Guayas, with Guayas accounting for the largest share (García, 2023; Ministerio de Salud Pública, 2023; Redacción 2023). By the end of that year, Guayas alone registered 314 cases, representing 47.3% of all national cases (Ministerio de Salud Pública Ecuador 2024). Seasonal floods during the coastal rainy season, frequently intensified by the El Niño–Southern Oscillation, likely contributed to this outbreak (Demoraes and D’Ercole 2001; Mwachui et al., 2015; Ortiz-Prado et al., 2023). Consistent with this seasonality, admissions in our cohort clustered in March and April, the peak of the rainy season. Historically, several studies on leptospirosis have been conducted in Guayas, including human studies reported in Guayaquil and surrounding areas (Yepez-plascencio et al., ; Chedraui Alvarez et al., 2001; Gelman et al., 2002; Gamboa et al., 2013).

**Figure 5:**
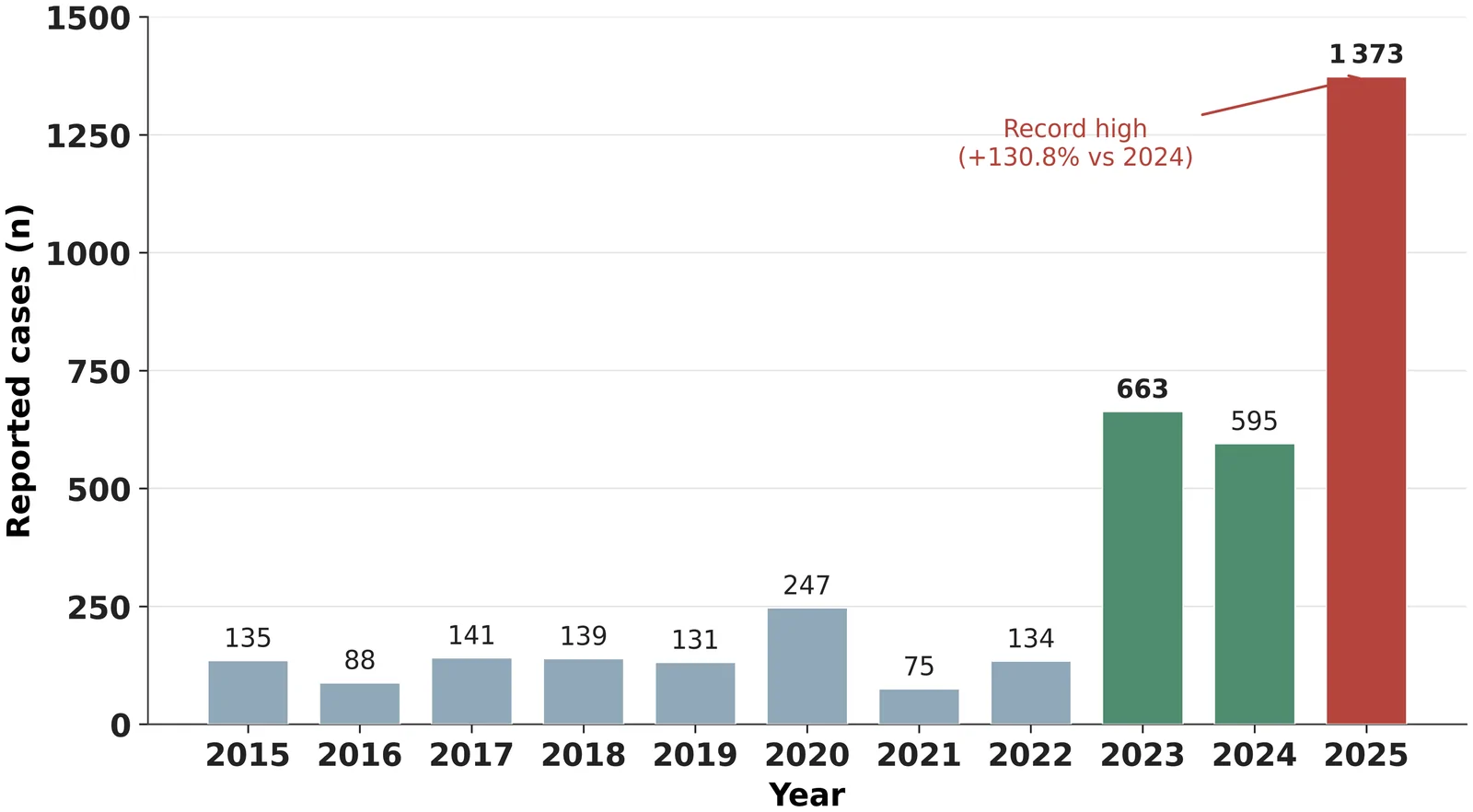
Annual leptospirosis cases reported in Ecuador, 2015–2025 (SIVE-ALERTA, National Directorate of Epidemiological Surveillance, Ministry of Public Health). Notifications reached a record high of 1, 373 cases in 2025, a 130.8% increase over 2024 (595) and more than double the previous peak of 2023 (663). Annual totals are provisional and subject to revision.

In our cohort, most cases occurred in men (58.3%), consistent with several studies. Calvopiña et al. analyzed leptospirosis in Ecuador over a 20-year period (2000–2020) and found a male predominance (65.7%) (Calvopiña et al., 2022). Studies from other countries show similar patterns, with male proportions of 79.2% in Brazil and 85.7% in Colombia (Tique et al., 2018; Galan et al., 2021). This male predominance aligns with broader Latin American trends, where 61–87.5% of cases occur in males, often linked to occupational and environmental exposures such as agriculture, livestock handling, or contact with contaminated water in both urban and rural settings (Vanasco et al., 2008; Daher et al., 2010; Echeverri-Toro et al., 2017; Parra Barrera et al., 2023). Globally, men account for approximately 80% of the total burden, corresponding to an estimated 2.33 million DALYs (Torgerson et al., 2015). The age distribution in our study also aligned with the literature: 85.5% of cases occurred in individuals aged 15–39 years, indicating a marked concentration of hospitalized cases among adolescents and younger adults, consistent with prior reports indicating that men aged 20–39 years are at highest risk (RR 2.4; 95% CI 0.7–4.1) (Costa et al., 2015). This pattern is further supported by Latin American data, where the mean age of affected patients ranges from 29 to 39 years, with peaks among young to middle-aged adults exposed to high-risk environments during rainy seasons or floods (Cedano et al., 2019; Lara et al., 2019; Menezes et al., 2024). Although not observed in our cohort, leptospirosis has been associated with cardiac involvement: myocarditis, while rare, has been described and may exceptionally progress to dilated cardiomyopathy, underscoring the importance of considering potential long-term sequelae in patients with cardiovascular symptoms (Izquierdo-Condoy et al., 2024).

Concurrent dengue IgM positivity was a notable finding, occurring in 22.9% of the full cohort (11/48) and 26.8% of patients tested for dengue (11/41). Leptospirosis and dengue are both important causes of febrile illness in tropical regions and frequently overlap temporally during the rainy season (Mishra et al., 2013; Ortiz-Prado et al., 2024; Shiddapur et al., 2024). Previous studies have reported simultaneous serological positivity or suspected co-infection in endemic settings (Brown et al., 2010; Sachu et al., 2018; Dhanashree and Shenoy, 2021). However, the interpretation of our finding requires caution. Because dengue status was based on IgM serology, concurrent IgM positivity does not by itself establish simultaneous acute infection and may also reflect recent dengue infection or other sources of serological uncertainty. Nevertheless, patients with dengue IgM positivity had a significantly longer median fever duration than dengue IgM-negative patients (19 vs 16 days; p=0.036), together with a trend toward longer hospital stay (9 vs 7 days; p=0.053). These exploratory findings reinforce the diagnostic complexity of overlapping febrile syndromes in endemic settings and support systematic evaluation of alternative or concurrent infections. In Latin American and Caribbean contexts, including Puerto Rico, overlapping febrile syndromes with complications such as renal failure have similarly been reported, underscoring the need for differential diagnostics in endemic areas (Adesiyun et al., 2011; Munoz-Zanzi et al., 2025).

One of the most striking findings was the prolonged duration of fever. More than half of the patients (55.3%) presented with fever lasting more than 15 days, and in two cases fever persisted for up to 60 days. The literature typically describes febrile periods of 3 to 7 days, often followed by a biphasic course in which fever and systemic complications may recur for up to 30 days (Reller et al., 2011; López-Cuenca and Álvarez Fernández, 2015). Cardoso et al. described a patient with a three-week history of fever (Cardoso et al., 2022), but we found no prior reports of leptospirosis with fever lasting as long as 60 days. This observation suggests that atypical or prolonged febrile presentations may occur in Ecuador, underscoring the need for closer clinical monitoring and further research into host–pathogen interactions in the local context. Across Latin American studies, fever is among the most common symptoms (85–99.5% of cases), often accompanied by myalgia (57–87.1%) and jaundice (58–94.5%), with severe complications such as renal impairment (up to 87% in some cohorts) contributing to variable mortality (0–26.1%) (Daher et al., 2010; Echeverri-Toro et al., 2017; Cedano et al., 2019; Lara et al., 2019).

One of the most striking observations was the prolonged documented duration of fever. More than half of patients (55.3%) had fever lasting more than 15 days, and two patients had febrile periods exceeding 45 days, including one reaching 60 days. Several factors may have contributed to the extended durations observed in our cohort, including delayed presentation or diagnosis, a biphasic disease course, concurrent febrile infections, and uncertainty inherent to serology-based diagnosis (Cedano et al., 2019; Rajapakse, 2022). Importantly, because this was a retrospective study, fever duration was derived from information documented in the medical record rather than prospectively monitored before hospitalization. Consequently, recall or documentation inaccuracies regarding symptom onset cannot be excluded. Delayed healthcare seeking or initial management as an undifferentiated febrile illness may also have increased the interval between symptom onset and hospital evaluation, although healthcare-access delays were not directly measured in this study. The extreme fever durations should therefore be interpreted cautiously and warrant confirmation in prospective studies.

Hypoalbuminemia was also particularly frequent among patients in whom albumin was measured. Its origin was likely multifactorial. Proteinuria was common, supporting a potential contribution from urinary protein loss, while liver-related laboratory abnormalities were also frequently observed (Haake and Levett, 2015). In addition, acute systemic infection may influence serum albumin through inflammatory responses, altered synthesis, redistribution, and changes in vascular permeability (Soeters et al., 2019). However, because albumin was measured in fewer than half of the cohort and markers allowing formal attribution to individual mechanisms were not systematically available, the relative contribution of hepatic dysfunction, renal protein loss, and systemic inflammation cannot be established from these data.

Anemia was another frequent finding and occurred in the context of several documented bleeding manifestations, including gastrointestinal bleeding, mucosal bleeding, hematuria, and positive fecal occult blood in a subset of tested patients. Nevertheless, bleeding cannot be assumed to explain all cases of anemia, as hematological abnormalities in leptospirosis may arise through multiple mechanisms, including blood loss, renal dysfunction, and, less commonly, hemolysis (Trowbridge et al., 1981). The retrospective dataset did not include a standardized etiological assessment of anemia, including systematic evaluation for iron deficiency, hemolysis, nutritional deficiencies, or pre-existing hematological disease. Accordingly, the anemia observed in this cohort should be regarded as potentially multifactorial and its specific mechanisms remain uncertain.

The empirical use of antibiotics in our cohort was heterogeneous, with ceftriaxone prescribed in most patients (89.6%) and doxycycline in fewer than a quarter (22.9%). This heterogeneity may reflect local practice variation and the lack of a standardized treatment protocol, underscoring the need for national evidence-based guidance. Our findings contribute novel evidence to the understanding of leptospirosis in Ecuador. This study represents the first clinical and epidemiological characterization of hospitalized patients in Durán, highlighting atypical features such as febrile episodes lasting up to 60 days—an observation not previously reported. The high prevalence of electrolyte disturbances, hematological abnormalities, and dengue co-infection underscores the diagnostic complexity of leptospirosis in tropical settings. Complementing these data, a recent report of a deadly outbreak in the Ecuadorian Amazon, where eight Indigenous children died and the mortality rate reached levels nearly 600 times higher than the national average, further illustrates the syndemic inequities shaping the burden of this neglected disease (Ortiz-Prado et al., 2025). Taken together, these findings, together with recent national surveillance trends, suggest increasing epidemiological complexity rather than proving a definitive shift in endemicity. In this context, leptospirosis in Ecuador may be expressing heterogeneous patterns, from clinically complex presentations in urban coastal populations to severe outbreaks in vulnerable Indigenous communities. These observations emphasize the need for strengthened surveillance, culturally adapted interventions, and a One Health approach.

## 5 Limitations

This study has several limitations. First, because the analysis was restricted to hospitalized patients, asymptomatic and mild infections managed in outpatient or community settings were not captured. In addition, the relatively small sample size and single-center design limit the generalizability of the findings to the broader population of Durán, Guayas province, or the Ecuadorian coast.

Second, the retrospective design relied on secondary clinical records that were not originally collected for research purposes. Consequently, some variables were incompletely documented or were not assessed using standardized definitions. In particular, fever duration was derived from the symptom history documented in the medical record rather than from prospective monitoring and may therefore be susceptible to recall and documentation bias. Likewise, a standardized mild, moderate, or severe disease classification had not been prospectively assigned; we therefore avoided retrospectively imposing a categorical severity classification that could introduce misclassification.

Third, leptospirosis diagnosis was based on positive anti-*Leptospira* IgM serology without confirmatory microscopic agglutination testing, PCR, paired serology, or serovar identification. Accordingly, the cohort should be interpreted as representing serologically supported rather than definitively microbiologically confirmed leptospirosis. Similarly, concurrent dengue infection was inferred from dengue IgM positivity, which cannot reliably distinguish simultaneous acute infection from recent infection or other sources of serological uncertainty. Findings related to concurrent dengue IgM positivity and the corresponding subgroup analyses should therefore be considered exploratory and hypothesis-generating. Other co-circulating causes of febrile illness, including Zika and chikungunya, were not systematically evaluated.

Fourth, the mechanisms underlying some of the frequent laboratory abnormalities could not be fully established. Although anemia occurred alongside gastrointestinal bleeding, mucosal bleeding, hematuria, and positive fecal occult blood in a subset of patients, systematic evaluation for iron deficiency, hemolysis, nutritional deficiencies, or pre-existing hematological disorders was not available. Similarly, although hypoalbuminemia coexisted with proteinuria and liver-related laboratory abnormalities, the relative contributions of urinary protein loss, hepatic dysfunction, systemic inflammation, and altered vascular permeability could not be determined.

Finally, the absence of systematic post-discharge follow-up precluded evaluation of long-term renal, hepatic, neurological, or cardiac sequelae. Socio-environmental determinants of transmission, healthcare-access delays, and other factors that may have influenced clinical presentation were also not directly measured. These limitations highlight the need for prospective, multicenter studies incorporating standardized severity assessment, confirmatory microbiological diagnostics, systematic evaluation of co-circulating infections, and longitudinal follow-up.

## 6 Conclusion

This study presents the first clinical–epidemiological characterization of hospitalized leptospirosis cases in Durán, Ecuador, describing a predominantly young, male population with frequent gastrointestinal and constitutional symptoms and high rates of laboratory abnormalities. We observed substantial overlap with dengue IgM positivity (≈23%) and an unusually prolonged febrile course—lasting up to 60 days in some patients—suggesting greater heterogeneity of clinical presentation than traditionally recognized. These findings, together with recent national surveillance trends, suggest increasing epidemiological complexity rather than proving a definitive shift in endemicity.

Our findings underscore urgent priorities: strengthening surveillance and diagnostic capacity, systematically evaluating co-infections, and implementing culturally adapted preventive strategies in high-risk populations. The development of national clinical practice guidelines for leptospirosis management is imperative, as such tools are currently lacking. In endemic regions such as the Coast and the Amazon, clinicians evaluating febrile patients should consider the possibility of overlapping or concurrent infections and use appropriate differential diagnostic testing whenever available. Future multicenter studies incorporating serovar typing, environmental and occupational exposures, and post-discharge outcomes are essential to refine clinical management and advance a One Health agenda aimed at reducing leptospirosis-related morbidity and mortality in Ecuador.

## Data Availability

The original contributions presented in the study are included in the article/supplementary material. Further inquiries can be directed to the corresponding author.
